# Neonatal and obstetric outcomes in diet- and insulin-treated women with gestational diabetes mellitus: a retrospective study

**DOI:** 10.1186/s12902-016-0136-4

**Published:** 2016-09-29

**Authors:** Sarah H. Koning, Klaas Hoogenberg, Kirsten A. Scheuneman, Mick G. Baas, Fleurisca J. Korteweg, Krystyna M. Sollie, Bertine J. Schering, Aren J. van Loon, Bruce H.R. Wolffenbuttel, Paul P. van den Berg, Helen L. Lutgers

**Affiliations:** 1 Department of Endocrinology, University of Groningen, University Medical Center Groningen, Hanzeplein 1, PO Box 30.001, 9700 RB Groningen, The Netherlands; 2 Department of Internal Medicine, Martini Hospital, Groningen, The Netherlands; 3 Department of Gynaecology and Obstetrics, Martini Hospital, Groningen, The Netherlands; 4 Department of Gynaecology and Obstetrics, University of Groningen, University Medical Center Groningen, Groningen, The Netherlands; 5 Department of Endocrinology, Medical Center Leeuwarden, Leeuwarden, The Netherlands

**Keywords:** Diet, Gestational diabetes mellitus, Insulin, Pregnancy outcomes, Treatment

## Abstract

**Background:**

To evaluate the neonatal and obstetric outcomes of pregnancies complicated by gestational diabetes mellitus (GDM). Screening and treatment – diet-only versus additional insulin therapy – were based on the 2010 national Dutch guidelines.

**Methods:**

Retrospective study of the electronic medical files of 820 singleton GDM pregnancies treated between January 2011 and September 2014 in a university and non-university hospital. Pregnancy outcomes were compared between regular care treatment regimens –diet-only versus additional insulin therapy- and pregnancy outcomes of the Northern region of the Netherlands served as a reference population.

**Results:**

A total of 460 women (56 %) met glycaemic control on diet-only and 360 women (44 %) required additional insulin therapy. Between the groups, there were no differences in perinatal complications (mortality, birth trauma, hyperbilirubinaemia, hypoglycaemia), small for gestational age, large for gestational age (LGA), neonate weighing >4200 g, neonate weighing ≥4500 g, Apgar score <7 at 5 min, respiratory support, preterm delivery, and admission to the neonatology department. Neonates born in the insulin-group had a lower birth weight compared with the diet-group (3364 vs. 3467 g, *p* = 0.005) and a lower gestational age at birth (*p* = 0.001). However, birth weight was not different between the groups when expressed in percentiles, adjusted for gestational age, gender, parity, and ethnicity. The occurrence of preeclampsia and gestational hypertension was comparable between the groups. In the insulin-group, labour was more often induced and more planned caesarean sections were performed (*p* = 0.001). Compared with the general obstetric population, the percentage of LGA neonates was higher in the GDM population (11.0 % vs.19.9 %, *p* = <0.001).

**Conclusions:**

Neonatal and obstetric outcomes were comparable either with diet-only or additional insulin therapy. However, compared with the general obstetric population, the incidence of LGA neonates was significantly increased in this GDM cohort.

## Background

Gestational diabetes mellitus (GDM) is a rising health problem worldwide and affects up to 14 % of all pregnancies, depending on the diagnostic criteria used and the population studied [1, 2]. GDM increases the risk of short-term and long-term adverse health outcomes for both mother and child, including neonatal and obstetric complications during childbirth and obesity and diabetes in later life [3–7].

Landmark studies have consistently shown that strict glycaemic control throughout pregnancy can effectively improve adverse health outcomes for mother and child [8–10]. Based on their results, new criteria for screening and treatment of GDM have been adopted in national and international guidelines. In the Netherlands, the Dutch Society of Obstetrics and Gynaecology guideline “Diabetes and Pregnancy” was revised in 2010 [11]. This guideline for the screening and treatment of GDM was largely based on the British National Institute for Health and Care Excellence (NICE) 2008 guidelines and the World Health Organization’s (WHO) diagnostic criteria (1999) for GDM, recommending to screen for GDM in high-risk women using the 2-h 75-g oral glucose tolerance test (OGTT) [12, 13]. This new guideline focused on a more active screening and treatment policy provided by “usual care” in the preceding years.

To date, the consequences of the guidelines on pregnancy treatment and outcomes have not been evaluated extensively. Moreover, as described in systematic reviews, most of the earlier studies only compared intensive treatment or any therapeutic intervention for GDM with usual obstetric care and made no distinction in pregnancy outcomes for diet-only treatment compared with insulin-treated women [14–18].

Hence, in this retrospective observational study we evaluated the neonatal and obstetric outcomes of pregnancies complicated by GDM. Screening and treatment – diet-only versus additional insulin therapy – were based on the 2010 national Dutch guidelines. We also compared these GDM outcomes with the general obstetric population in the Northern region of the Netherlands.

## Methods

### Study design and population

This retrospective observational cohort study of women with GDM was conducted in two hospitals in the North of the Netherlands, University Medical Center Groningen and non-university Martini Hospital Groningen. Those centers adopted a joint protocol on screening and treatment of GDM in January 2011 after revision of the Dutch guideline in 2010 [11]. The electronic medical files of all women with a diagnosis of GDM, who visited the outpatient clinic of both hospitals between January 2011 and September 2014, were eligible for inclusion in the study.

Pregnant women were tested for GDM if they had risk factors for GDM according to the Dutch guideline or signs suggestive of GDM (like foetal macrosomia and/or polyhydramnion). These GDM risk factors were: previous GDM, pre-gestational body mass index (BMI) ≥30 kg/m^2^, previous infant weighing ≥4500 g at birth, first-degree relative with type 2 diabetes, ethnic origin (South-Asian, Hindu, Afro-Caribbean, Middle-Eastern, Morocco, and Egypt), history of intrauterine fetal death, and history of polycystic ovary syndrome. Pregnant women with GDM risk factors were routinely screened for GDM at 24–28 weeks of gestation by their midwife’s office care, or by their gynaecologist in secondary care. Women with previous GDM were screened at 16–18 weeks of gestation and when the results were negative, this was repeated in week 24–28 of gestation.

A 75-g OGTT was used for the screening of GDM. GDM was diagnosed when fasting plasma glucose was ≥7.0 mmol/l and/or 2-h value ≥7.8 mmol/l after the 75-g glucose load, according to the diagnostic criteria of the WHO (1999) [12]. In addition, GDM was diagnosed without an OGTT if fasting glucose was >7.0 mmol/l or a random glucose was >11.1 mmol/l.

**Fig. 1:**
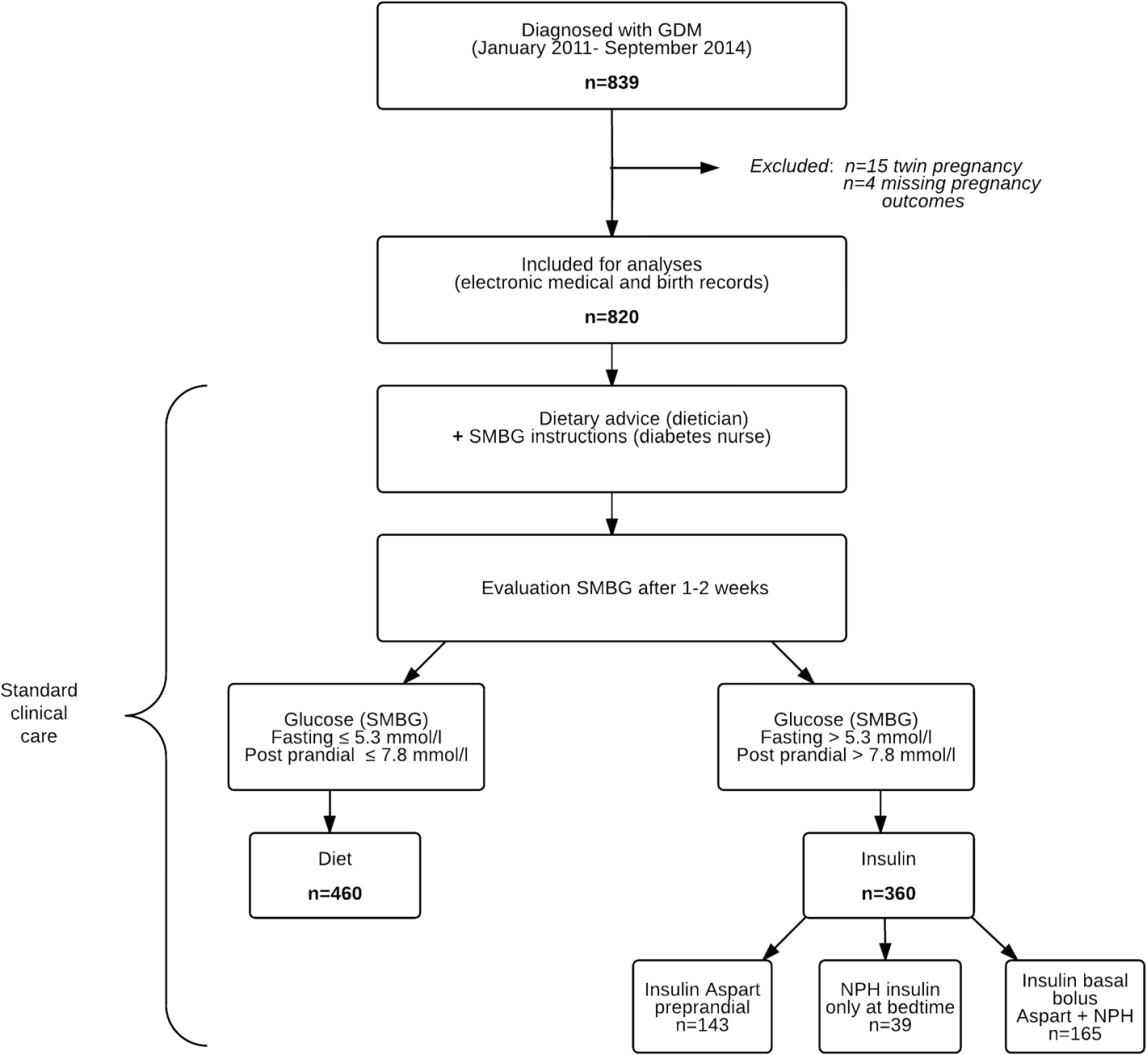
Flow-chart of the study design. GDM, Gestational Diabetes Mellitus; NPH, Neutral Protamine Hagedorn; SMBG; Self-Monitoring Blood Glucose

In total 839 women were diagnosed with GDM and referred to the diabetes outpatient clinic for treatment. For the present analysis, women with twin pregnancy (*n* = 15) or missing pregnancy outcomes (*n* = 4) were excluded (Fig. 1). This study is conducted in accordance with the guidelines of the Declaration of Helsinki and Good Clinical Practice. The study has been exempted for approval according to the Medical Research Involving Human Subjects Act [19]. This report is based on patient data acquired during care-as-usual, the data has been analyzed retrospectively and all the requirements for patient anonymity are in agreement to the regulations of the ethical committee of both hospitals for publication of patient data. According to this and the Dutch law Medical Research with Human Subjects, no approval from an ethics committee is necessary.

### GDM management

The first step in the management of women with GDM was dietary advice by a dietician, which included advice about carbohydrate intake and carbohydrate distribution. Women were also instructed to measure fasting and 1-h postprandial blood glucose levels every day. Blood glucose levels were reviewed after 1–2 weeks. Women with fasting blood glucose level >5.3 mmol/l and/or post prandial blood glucose level >7.8 mmol/l received additional insulin therapy to obtain blood glucose values below these treatment goals. Insulin was commenced when two blood glucose results at the same moment of the day were elevated despite dietary intervention. Insulin treatment regimens were: long-acting insulin only, prandial short-acting insulin or a combination of both (basal-bolus regimen), depending on the individual glycaemic profile. In both centres short-acting insulin analogues and Neutral Protamine Hagedorn (NPH) insulin were used in GDM treatment.

Women were intensively followed with regular e-mail and/or telephone contact, at least weekly, in order to assist them to achieve and maintain the glycaemic targets. Every 3–4 weeks or on request if indicated, women visited the diabetes and obstetric outpatient clinics. If applicable based on self-monitoring of the blood glucose values, diet was adjusted and insulin dose increased to maintain blood glucose levels within the target range.

Fetal growth was evaluated by ultrasonography, performed every 4 weeks by trained obstetricians. In both centres labour was induced at or around 38 weeks in women on insulin therapy with taking blood glucose control as well as fetal growth into consideration. Labour was also induced for non-GDM related maternal or fetal indications, for instance gestational hypertension or preeclampsia. The diet- and insulin-treated women were both followed to the date of delivery and were discussed every 3 weeks in a multidisciplinary consultation in the University Medical Center Groningen. In the Martini Hospital multidisciplinary consultation occurred immediately after the outpatient clinic visit.

### Clinical data collection

All data were collected from electronic medical- and birth records. Ethnicity was labeled in five categories: Caucasian, Asian (Indian or South-East Asian), African-American, Mediterranean (Hispanic, Middle-Eastern, North-African or South-American), and unknown. Chronic hypertension was defined as a systolic blood pressure (SBP) ≥140 mmHg, a diastolic blood pressure (DBP) ≥90 mmHg on two occasions at least 4 h apart or the use of blood-pressure lowering drugs, before pregnancy. Family history of diabetes was defined as having a first degree relative who had type 2 diabetes. HbA1c values were measured by standardized HPLC method on a Tosoh G8 system (Tosoh, Tokyo, Japan), considering 22–42 mmol/mol (4.2–6.0 %) as normal. The HbA1c values were measured at the time of GDM diagnosis within 1 week after the OGTT.

### Neonatal outcomes

Neonatal outcomes were a composite outcome of perinatal complications (still birth/neonatal death, birth trauma (shoulder dystocia, fracture of humerus or clavicle, brachial plexus injury), neonatal hypoglycaemia and neonatal hyperbilirubinaemia), gestational age at birth, birth weight, neonate weighing >4000–4499 g, neonate weighing >4200 g, neonate weighing ≥4500 g, large for gestational age (LGA) (defined as birth weight above the 90^th^ percentile, adjusted for gestational age, gender, parity, and ethnicity [20]), small for gestational age (SGA) (defined as birth weight below the 10^th^ percentile, adjusted for gestational age, gender, parity, and ethnicity [20]), Apgar score <7 at 5 min, need for respiratory support, preterm delivery (defined as delivery before 37 completed weeks of gestation), and admission to the neonatology department.

The presence of neonatal hypoglycaemia (occurring >2 h after birth) was defined as a blood glucose level <2.6 mmol/l or treatment with a glucose infusion [11]. Neonatal hyperbilirubinaemia was recorded when the infant was treated with phototherapy after birth or admission at the neonatology department for this reason. Respiratory support was defined as the need for supplemental oxygen or continuous positive airway pressure after birth.

### Obstetric outcomes

The obstetric outcomes were: induction of labour, delivery type (spontaneous, instrumental (forceps or vacuum extraction), planned caesarean section and secondary caesarean section), gestational hypertension, and preeclampsia.

Gestational hypertension was defined as a SBP ≥140 mmHg and/or a DBP ≥90 mmHg, with no evidence of pre-existing hypertension and the absence of proteinuria. Preeclampsia was defined as a combination of gestational hypertension and proteinuria (≥300 mg/24-h) and included eclampsia and ‘Hemolysis Elevated Liver enzymes and Low Platelets’ (HELLP)-syndrome.

For comparison, the general obstetric population in the Northern region of the Netherlands and their registered neonatal and obstetric outcomes during the period 2011–2013 served as a reference population screened with the same guidelines, these data were provided from the Dutch Perinatal Registry and the Municipal Health Service Groningen. Data of the general obstetric population were available for the following outcomes: still birth/neonatal death, neonate weighing >4000–4499 g, neonate weighing ≥4500 g, LGA, SGA, and Apgar score <7 at 5 min.

### Statistical analyses

Continuous data are presented as mean ± standard deviation (SD) or as median and inter quartile range [IQR] in case of skewed distribution. Categorical data are presented as number and percentage. Differences between the groups were tested using Student’s unpaired *t*-test for continuous data or Mann-Whitney *U* Test in case of skewed distribution. For categorical data Chi-square or Fisher’s exact test were used.

All *P*-values are two-tailed, and *P*-values <0.05 were considered statistically significant. All analyses were conducted with the use of the statistical package IBM SPSS (version 22.0. Armonk, NY: IBM Corp).

## Results

The maternal characteristics are summarized in Table 1. Of the 820 women with GDM, 460 women (56.1 %) met the glycaemic goals on diet-only and 360 women (43.9 %) required additional insulin therapy. Of the women who required additional insulin therapy, 143 women (40 %) received trice daily pre-prandial short-acting insulin, 165 women (46 %) received basal-bolus insulin therapy and 39 women (11 %) received long-acting insulin therapy to achieve the glycaemic targets (*n* = 13 missing data on type of insulin). The median insulin dose was 22 U/day; IQR 12–42 U/day. All women were monitored and treated similarly and achieved good glycaemic control.﻿To establish glycaemic control third trimester HbA1c values were evaluated (week 32-36 of gestation). For a small sample of women (* n *= ﻿212) the HbA1c values were﻿ also measured in third trimester of their pregnan﻿cy. The median HbA1c values were higher in the insulin-g﻿roup (*n* = 125; median 5.7 % (39 mmol/mol), IQR 5.4-6.0 % (36-42 mmol/mol)) compared with the diet-group (*n *= 87; median 5.5 % (37 mmol/mol), IQR 5.3-5.6 % (34-38 mmol/mol)). The women in the insulin-group were slightly older and were more often overweight (BMI ≥25 kg/m^2^). In addition, multiparity was higher in the insulin-group and a higher proportion had a previous GDM, previous infant weighing ≥4500 g at birth, and a family history of diabetes. The median fasting glucose level and 2-h glucose level after a 75-g OGTT were higher in the insulin-group compared with the diet-group. In the whole cohort, GDM diagnosis was based only on the fasting glucose in 1 % during the OGTT, and 91 % tested positive only on the 2-h value. In total 9685 women (with a mean maternal age of 30.9 ± 4.9) and 9854 neonates of the Northern region of the Netherlands served as a reference population.

**Table 1:** Clinical characteristics according to the treatment groups of 820 women with gestational diabetes mellitus

		Treatment groups		
Characteristics	Overall	Diet	Insulin	*P*-value*
*N* (%)	820	460 (56.1)	360 (43.9)	
Age (years)	32.0 ± 5.1	31.6 ± 4.9	32.6 ± 5.2	0.010
Ethnicity, *n* (%)				NS
Caucasian	658 (80.2)	377 (82.0)	281 (78.1)	
Asian	55 (6.7)	35 (7.6)	20 (5.6)	
African-American	35 (4.3)	18 (3.9)	17 (4.7)	
Mediterranean	57 (7.0)	22 (4.8)	35 (9.7)	
Unknown	15 (1.8)	8 (1.7)	7 (1.9)	
Family history of DM, *n* (%)	326 (39.8)	156 (33.9)	170 (47.2)	<0.001
History of PCOS, *n* (%)	40 (4.9)	24 (5.2)	16 (4.4)	NS
Previous GDM, *n* (%)	86 (10.5)	25 (5.4)	61 (16.9)	<0.001
Previous infant weighing ≥4500 g at birth, *n* (%)	90 (11.0)	35 (7.6)	55 (15.3)	<0.001
History of IUFD, *n* (%)	16 (2.0)	5 (1.1)	11 (3.1)	0.043
History of spontaneous abortion, *n* (%)	223 (27.2)	113 (24.6)	110 (30.6)	NS
Chronic hypertension, *n* (%)	37 (4.5)	15 (3.3)	22 (6.1)	NS
Smoking during pregnancy, *n* (%)	81 (9.9)	42 (9.1)	39 (10.8)	NS
Multigravida, *n* (%)	564 (68.8)	285 (62.0)	279 (77.5)	<0.001
Parity, *n* (%)				<0.001
0	333 (40.6)	223 (48.5)	110 (30.6)	
1–2	436 (53.2)	216 (47.0)	220 (61.1)	
> 2	51 (6.2)	21 (4.6)	30 (8.3)	
Pre-gestational BMI, *n* (%) ^b^				<0.001
< 25 kg/m^2^	260 (32.7)	173 (38.7)	87 (25.0)	
25–29.9 kg/m^2^	231 (29.1)	126 (28.2)	105 (30.2)	
≥ 30 kg/m^2^	304 (38.2)	148 (33.1)	156 (44.8)	
Pre-gestational BMI (kg/m^2^)	27.7 [24.0–31.9]	26.9 [23.3–31.4]	29.2 [25.0–33.4]	<0.001
Weight gain mother (kg) ^a^	8.0 [4.0–12.0]	9.0 [5.0–13.0]	7.0 [3.0–11.0]	<0.001
Indication for OGTT, n (%) ^c^				0.010
Risk factors	523 (66.2)	275 (62.4)	248 (71.1)	
Signs	267 (33.8)	166 (37.6)	101 (28.9)	
Fasting glucose level (mmol/l)	5.0 [4.6–5.5]	4.8 [4.5–5.2]	5.3 [4.9–5.9]	<0.001
2-h glucose level after a 75-g OGTT (mmol/l)	8.6 [8.1–9.4]	8.5 [8.0–9.1]	8.8 [8.2–9.7]	<0.001
Abnormal value only on fasting glucose level, *n* %	8 (1.0)	3 (0.7)	5 (1.5)	NS
Abnormal value only on 2-h glucose level, *n* %	746 (91.0)	440 (95.7)	306 (85)	0.003
Gestational age at time of OGTT (wks)	27.9 [25.9–30.7]	28.4 [26.7–32.3]	27.1 [24.4–29.3]	<0.001
HbA1c ^d^				
mmol/mol	37 [34–40]	37 [34–39]	38 [36–42]	<0.001
%	5.5 [5.3–5.8]	5.5 [5.3–5.7]	5.6 [5.4–6.0]	

Data are expressed as mean ± SD, median [IQR], or proportion n (%)

*Abbreviations*: *BMI* Body Mass Index, *DM* Diabetes Mellitus, *﻿GDM﻿ *Gestational Diabetes Mel﻿litus, *IUFD* Intrauterine Fetal Death, *PCOS* Polycystic Ovary Syndrome, *OGTT* Oral Glucose Tolerance Test, *HbA1c* Haemoglobin A1c, *wks* weeks, *g* gram, *NS* not significant

* *P*-values were based on Student’s unpaired *t*-test (non-skewed continuous variables), Mann-Whitney *U*-Test (skewed continuous variables) or chi-square test (categorical variables)

^a^Weight gain from pre-pregnancy weight to first visit

^b^ *N* = 795 due to missing data on weight or height

^c^ *N* = 790 due to missing data on indication of OGTT

^d^ *N* = 643 due to missing data. The HbA1c values were measured at the time of GDM diagnosis within 1 week after the OGTT

### Neonatal outcomes

Table 2 shows the neonatal outcomes. Between the treatment groups, there were no significant differences in neonatal outcomes with respect to the perinatal complications (*p* = 0.221). Although the frequency of neonatal hypoglycaemia and neonatal hyperbilirubinaemia tended to be higher in the insulin-group, these differences were not statistically significant. For the variable neonatal hypoglycaemia, neonates born <37 weeks of gestation with neonatal hypoglycaemia were excluded (*n* = 7). Neonates born to women in the insulin-group had a significantly lower birth weight (3364 vs. 3467 g, *p* = 0.005) and a lower gestational age at birth (38.1 vs. 38.6 weeks, *p* = 0.001) compared with neonates born to women in the diet-group. Moreover, the frequency of neonates weighing >4000–4499 g were higher in the diet-group compared with the insulin group (14.0 % vs. 8.8 % *p* = 0.009). However, birth weight was not different between the groups when expressed in percentiles (*p*= > 0.05), adjusted for gestational age, parity, ethnicity, and gender. For the variable birth weight, neonates with extreme prematurity (born <28 weeks) were excluded (*n* = 3), to remove the potential bias for extreme low birth weight. There were no significant differences between the two groups with respect to LGA, SGA, neonate weighing >4200 g, neonate weighing ≥4500 g, Apgar score <7 at 5 min, need for respiratory support, preterm delivery, and admission to the neonatology department.

**Table 2:** Neonatal outcomes according to gestational diabetes mellitus treatment groups

		Treatment groups				
Outcome variable	Overall	Diet	Insulin	*P*-value*	General obstetric population	*P*-value**
*N* (%)	820	460 (56.1)	360 (43.9)		9854 ^a^	
Perinatal complications, *n* (%) ^b^	75 (9.1)	36 (7.8)	39 (10.8)	NS		
Still birth/neonatal death, *n* (%) ^c^	2 (0.2)	0 (0.0)	2 (0.6)	NS	89 (0.9)	0.048
Birth trauma, *n* (%)	30 (3.7)	19 (4.1)	11 (3.1)	NS		
Hypoglycaemia, *n* (%) ^d^	28 (3.4)	12 (2.6)	16 (4.5)	NS		
Hyperbilirubinaemia, *n* (%)	15 (1.8)	5 (1.1)	10 (2.8)	NS		
Birth weight (g) ^e^	3422 ± 522	3467 ± 522	3364 ± 517	0.005		
Infants >4000–4499 g, *n* (%)	96 (11.7)	63 (14.0)	32 (8.8)	0.009	1207 (12.2)	0.350
Infants >4200 g, *n* (%)	40 (4.9)	25 (5.4)	15 (4.2)	NS		
Infants ≥4500 g, *n* (%)	10 (1.2)	8 (1.7)	2 (0.6)	NS	218 (2.2)	0.059
Birth weight percentiles, *n* (%) ^f^				NS		
< 10^th^ percentile	27 (3.3)	19 (4.1)	8 (2.2)			
10–20^th^ percentile	44 (5.4)	20 (4.3)	24 (6.7)			
20–50^th^ percentile	202 (24.6)	109 (23.7)	93 (25.8)			
50–90^th^ percentile	384 (48.6)	214 (46.5)	170 (47.2)			
90–95^th^ percentile	67 (8.2)	46 (10.0)	21 (5.8)			
> 95^th^ percentile	96 (11.7	52 (11.3)	44 (12.2)			
Large for gestational age, *n* (%) ^g^	163 (19.9)	98 (21.3)	65 (18.1)	NS	1082 (11.0)	<0.001
Small for gestational age, *n* (%) ^h^	27 (3.3)	19 (4.1)	8 (2.2)	NS	788 (8.0)	<0.001
Gestational age at birth (wks)	38.3 [38.0–39.0]	38.6 [38.0–39.6]	38.1 [38.0–38.4]	<0.001		
Apgar <7 at 5 min, *n* (%)	27 (3.3)	17 (3.7)	10 (2.8)	NS	92 (2.0)	0.009
Respiratory support, *n* (%)	26 (3.2)	16 (3.5)	10 (2.8)	NS		
Preterm delivery, *n* (%)				NS		
< 28 weeks	3 (0.4)	1 (0.2)	2 (0.6)			
28–32 weeks	5 (0.6)	2 (0.4)	3 (0.8)			
32–37 weeks	44 (5.4)	25 (5.4)	19 (5.3)			
Admission neonatology, *n* (%)	121 (14.8)	61 (13.3)	60 (16.7)	NS		

Data are expressed as mean ± SD, median [IQR], or proportion n (%)

*Abbreviations*: *g* grams, *wks* weeks, *NS* not significant

* *P*-values were based on Student’s unpaired *t*-test (non-skewed continuous variables), Mann-Whitney *U*-Test (skewed continuous variables) or chi-square test (categorical variables)

** *P*-values for the GDM population (overall) and general obstetric population were based on chi-square test

^a^ In total *n* = 9685 mothers with a mean age of 30.9 ± 4.9. Not all of the neonatal and obstetric outcomes in the general population were well reported and this has resulted in lack of information for some neonatal outcomes

^b^ Perinatal complications included the following: still birth/neonatal death, birth trauma (shoulder dystocia, fracture of humerus or clavicula), hypoglycaemia, and hyperbilirubinaemia

^c^ One still birth was associated with gestational diabetes and the other still birth was associated with a congenital heart defect

^d^ Hypoglycaemia was defined as neonates without prematurity (born <37 weeks of gestation). There were *n* = 7 neonates with hypoglycaemia and prematurity excluded

^e^ Mean birth weight was calculated after exclusion of neonates with extreme prematurity (born <28 weeks of gestation). There were *n* = 3 neonates with extreme prematurity (178 days, 185 days, and 195 days)

^f^ Birth weight in percentiles were adjusted for gestational age, gender, parity, and ethnicity

^g^ Large for Gestational Age was defined as a birth weight above the 90^th^ percentile, adjusted for gestational age, gender, parity, and ethnicity

^h^ Small for Gestational Age was defined as birth weight below the 10^th^ percentile, adjusted for gestational age, gender, parity, and ethnicity

In comparison, the percentage of LGA neonates with a birth weight >90^th^ percentile was significantly higher in the GDM population (19.9 %) compared to the general obstetric reference population (11.0 %) (*p* = <0.001). Further, the percentage of SGA neonates with a birth weight <10^th^ percentile was significantly lower in the GDM population (3.3 %) compared to the general obstetric reference population (8.0 %) (*p* = <0.001). Apgar score <7 at 5 min was significantly lower in the general obstetric reference population (2.0 %) compared with the GDM population (3.3 %) (*p* = 0.009). There were no differences between the GDM population and the general obstetric reference population with respect to infants weighing >4000–4499 g and infants weighing ≥4500 g.

### Obstetric outcomes

The obstetric outcomes are shown in Table 3. Labour was induced more frequently in the insulin-group. However, insulin therapy was one of the indications to induce labour in both hospitals at or around 38 weeks. There were significantly more planned caesarean sections in the insulin-group compared with the diet-group (*p* = 0.001) while secondary caesarean sections were comparable (*p* = 0.335). There were more instrumental vaginal deliveries in the diet-group (*p* = 0.052). There were no differences in occurrence of preeclampsia and gestational hypertension between the two groups.

**Table 3:** Obstetric outcomes according to gestational diabetes mellitus treatment groups

		Treatment groups		
Outcome variable	Overall	Diet	Insulin	*P*-value*
*N* (%)	820	460 (56.1)	360 (43.9)	
Induction of labour, *n* (%)	533 (65.0)	271 (58.9)	262 (72.8)	NA ^a^
Delivery type, *n* (%)				
Spontaneous	561 (68.4)	317 (68.9)	244 (67.8)	NS
Instrumental ^b^	67 (8.2)	46 (10.0)	21 (5.8)	NS
Caesarean section	99 (12.1)	60 (13.0)	39 (10.8)	NS
Planned caesarean section	93 (11.3)	37 (8.0)	56 (15.6)	0.001
Preeclampsia, *n* (%) ^c^	28 (3.4)	16 (3.5)	12 (3.3)	NS
Gestational hypertension, *n* (%)	75 (9.1)	43 (9.3)	32 (8.9)	NS

Data are expressed as proportion n (%)

*Abbreviations*: *NS* not significant, *NA* not applicable

* *P*-values were based on chi-square test for categorical variables

^a^ Not applicable, insulin therapy is one of the indications to induce labour in GDM pregnancy at or around 38 weeks of gestation

^b^ Instrumental is defined as forceps and vacuum extraction

^c^ Preeclampsia included eclampsia (*n* = 1) and Hemolysis Elevated Liver enzymes and Low Platelets (HELLP) syndrome (*n* = 1)

## Discussion

In this retrospective observational cohort study of 820 singleton GDM pregnancies treated according the revised national guideline on systematic screening and treatment of GDM, we found a higher incidence of LGA neonates of approximately 20 % compared with 11 % in the general obstetric population in the Northern region of the Netherlands. However, there were no major differences in neonatal and obstetric outcomes between women treated with diet-only and those who needed additional insulin therapy.

A recent large population-based study investigated the pregnancy outcomes complicated by pre-existing diabetes and GDM in Alberta, Canada [21]. Compared with our GDM population we found comparable results for the following adverse pregnancy outcomes, preeclampsia, stillbirth, admission to the neonatology department, and Apgar score below 7 at 5 min. In the Canadian study there was a much higher proportion of caesarean sections performed in the GDM pregnancies compared with our study (36.9 % vs. 12.1 %). Furthermore, the percentage LGA infants was lower in the Canadian GDM population (15.3 % vs. 19.9 %) and they found a higher percentage of SGA (9.4 % vs. 3.3 %) infants compared with our study [21].

Five systematic reviews have summarized the studies specifically on the effect of treatment of GDM on pregnancy outcomes [14–18]. They included studies that compared intensive treatment – including diet modification, glucose monitoring and/or insulin – or any therapeutic intervention of GDM with usual obstetric care in GDM women. It was shown that intensive treatment of GDM reduced the risk of preeclampsia, shoulder dystocia, and macrosomia. In line with our findings, these reviews reported a low incidence of serious adverse outcomes, like mortality and birth trauma.

In contrast with the landmark trials on treatment of GDM, the number of LGA neonates in our study was relatively high [8, 9]. In the Australian Carbohydrate Intolerance Study (ACHOIS) the prevalence of LGA neonates was 13 % in the treatment group and in the study by Landon et al. it was 7.1 % [8, 9]. This discrepancy in the prevalence of LGA neonates can be possibly explained by the differences in diagnostic criteria for GDM. Especially, the study by Landon et al., included women with milder glucose intolerance using the following cut-off values: fasting value <5.3 mmol/l; 1-h value ≥10.0 mmol/l; 2-h value ≥8.6 mmol/l; and 3-h value ≥7.8 mmol/l after a 100-g OGTT [8]. Compared with our study, they included women who had slightly lower fasting glucose value but higher post GTT value. Furthermore, different definitions for LGA were used between the studies. In the Landon study the percentage neonates with a birth weight >4000 g was almost similar with the percentage observed in our GDM population. However, in the Landon study there was a small difference between the proportion neonates with a birth weight >4000 g and the proportion LGA neonates, as they did not correct for gender, ethnicity and parity [8]. In our study LGA was defined as a birth weight above the 90^th^ percentile, specifically adjusted for gender, parity and ethnicity [20]. Therefore, we found a larger difference between neonates weighing >4000 g and LGA neonates.

In 2010 the International Association of the Diabetes and Pregnancy Study Groups (IADPSG) and in 2013 the WHO adopted new diagnostic criteria for GDM [22, 23]. The WHO 2013/IADPSG diagnostic criteria for GDM recommends the following 75-g OGTT glycaemic thresholds: fasting value ≥5.1 mmol/l; 1-h ≥10.0 mmol/l; and a 2-h value ≥8.5 mmol/l [22, 23]. In our study the WHO 1999 [12] diagnostic criteria for GDM were used, with a much higher fasting glucose threshold. However, the screening and diagnostic criteria for GDM are inconsistent across Europe [24]. The recently revised British NICE guideline 2015 recommends the following alternative 75-g OGTT glycaemic thresholds: fasting value ≥5.6 mmol/l; 2-h value ≥7.8 mmol/l [25]. The 2-h glucose level of the NICE guideline corresponds with our guideline for the diagnosis of GDM, only the NICE guideline proposed a lower fasting glucose value. The NICE guideline proposed these thresholds, because of the treatment costs and the limited evidence for treating at lower diagnostic thresholds [25]. Nevertheless, a recent study comparing the outcomes among the IADPSG criteria and NICE 2015 criteria, demonstrated that women who test positive for GDM according to the IADPSG criteria but negative for the NICE 2015 criteria, had the highest risk of having infants with LGA. In other words, the IADPSG criteria identified women at risk of LGA who may benefit from treatment, but these women were unidentified and not treated with the WHO 1999 [12] guideline and NICE [25] guideline [26]. A similar problem of too high cut-off value for fasting blood glucose may hamper the Dutch guideline.

A reduction in the proportion of LGA neonates is an important treatment target in GDM, since LGA neonates apart from the risk of obstetrical complications are possible more likely to develop obesity and diabetes in early adulthood and later life [3, 4]. The more stringent IADPSG glycaemic thresholds may contribute as they have been shown to accurately identify women at risk of delivery LGA neonates [22, 26]. The timing of the diagnosis and intervention of GDM is another factor of concern. GDM screening is advised between 24 and 28 weeks of gestation. In our study the median gestational age at GDM diagnosis ranged from 26 to 31 weeks, in a considerable proportion of women, intervention was started late in pregnancy. The OGTT could be scheduled more strictly at week 24 or even before week 24. One recent study [27] has shown that despite early testing and treatment, early GDM diagnosis (<24 weeks) in high-risk women was associated with adverse pregnancy outcomes, including LGA neonates. The study indicates that early identification in high-risk GDM women is important to improve the pregnancy outcomes [27]. However, screening for GDM before 24 weeks of gestation would increase the amount of false negative OGTT’s.

The neonates born in the diet-group and insulin-group were more likely to be LGA compared with the general obstetric population in the Northern region of the Netherlands. It has been shown that LGA is not always a consequence of hyperglycaemia, other risk factors such as pre-pregnancy overweight/obesity, maternal weight gain during pregnancy, and maternal age increases the risk of having a LGA neonate [28–31]. The women in this GDM cohort were older compared with the general obstetric population and the percentage of pre-pregnancy overweight women in our present cohort was high, almost 70 %, but not higher than the Landon study [8]. However, a recent meta-analysis has shown that lifestyle intervention in obese pregnant women reduces maternal weight gain, but without differences in LGA or macrosomia [32].

The main strength of this study is that it evaluated the results of the implementation of the in 2010 introduced guideline in a large GDM population. It further focuses on outcomes in diet-only and insulin-treated women. Therefore the study is indicative for routine clinical practice and makes the results more applicable to real-life clinical care. An additional strength is the comparison with data from the general obstetric population in the same region of the Netherlands.

A theoretical limitation of this study is the retrospective design. This may have resulted in lack of information from variables in existing medical and birth records, however most of the medical and birth records in our study were complete. A further limitation is that our national guideline uses the “old” WHO 1999 diagnostic criteria for GDM which differ greatly from the new WHO 2013 criteria [12, 23]. On the other hand, for the treatment of GDM we use the new stringent international glucose targets to obtain glycaemic control in GDM pregnancies. At last, not all the neonatal and obstetric outcomes in the general population are available from public dataset with sufficient detail and this has resulted in lack of information for some neonatal and obstetric outcomes.

Our current GDM guideline on systematic screening and stringent treatment of GDM was successful in achieving a low incidence of birth complications comparable to the literature, but the percentages of LGA neonates was significantly higher in GDM compared to other international GDM studies, and to the general obstetric population. This has practical implications for the Dutch clinical guideline for GDM that is not optimal at this moment for reducing LGA neonates. Adopting stricter diagnostic criteria should be considered in the next revision of the Dutch guideline, the current criteria is likely not stringent enough. There is more evidence from other countries that the more stringent criteria is associated with significant improvements in pregnancy outcomes, including lowering the frequency of LGA neonates [33]. Furthermore, we agree with Benhalima et al. who ultimately recommend the use of the same diagnostic criteria for GDM across Europe [24].

## Conclusions

In summary, in this GDM population screened and treated according to the 2010 national guideline, we found an elevated percentage of LGA neonates compared with the general obstetric reference population. Although the incidence of severe pregnancy outcomes was low. We found no major differences in neonatal and obstetric outcomes between GDM women treated with diet-only or additional insulin. Despite the progress in screening and treatment of GDM, we suggest an adjustment of the diagnostic criteria is needed to further improve GDM outcomes, especially to reduce the risk of LGA neonates for obesity and diabetes later in life.

## Abbreviations

ACHOIS: Australian Carbohydrate Intolerance Study
BMI: Body mass index
DBP: Diastolic blood pressure
GDM: Gestational diabetes mellitus
HELLP: Hemolysis Elevated Liver enzymes and Low Platelets
IADPSG: International Association of the Diabetes and Pregnancy Study Groups
IQR: Inter quartile range
LGA: Large for gestational age
NICE: National Institute for Health and Care Excellence
NPH: Neutral Protamine Hagedorn
OGTT: Oral glucose tolerance test
SBP: Systolic blood pressure
SD: Standard deviation
SGA: Small for gestational age
WHO: World Health Organization
